# B Cells Modulate Mucosal Associated Invariant T Cell Immune Responses

**DOI:** 10.3389/fimmu.2013.00511

**Published:** 2014-01-07

**Authors:** Rosangela Salerno-Goncalves, Tasmia Rezwan, Marcelo B. Sztein

**Affiliations:** ^1^Department of Pediatrics, Center for Vaccine Development (CVD), University of Maryland School of Medicine, Baltimore, MD, USA

**Keywords:** MAIT cells, B cells, human, gut, commensals

## Abstract

A common finding when measuring T cell immunity to enteric bacterial vaccines in humans is the presence of background responses among individuals before immunization. Yet the nature of these background responses remains largely unknown. Recent findings show the presence in uninfected individuals of mucosal associated invariant T (MAIT) cells that mount broad spectrum immune responses against a variety of microorganisms including *Mycobacterium tuberculosis* and enteric bacteria such as *Escherichia coli* and *Salmonella*. Therefore, we investigated whether MAIT immune responses to intestinal bacteria might account for the background responses observed before immunization. Here we measured MAIT immune responses to commensal and enteric pathogenic bacteria in healthy individuals with no history of oral immunization with enteric bacteria. We found that MAIT cells were activated by B cells infected with various bacteria strains (commensals and pathogens from the *Enterobacteriaceae* family), but not by uninfected cells. These responses were restricted by the non-classical MHC-related molecule 1 (MR1) and involved the endocytic pathway. The quality of these responses (i.e., cytokine profile) was dependent on bacterial load but not on the level expression of MR1 or bacterial antigen on B cell surface, suggesting that a threshold level of MR1 expression is required to trigger MAIT activation. These results provide important insights into the role of B cells as a source of antigen-presenting cells to MAIT cells and the gut immune surveillance of commensal microbiota.

## Introduction

A common finding when measuring T cell immune responses in humans to enteric bacterial vaccines is the presence of background responses among individuals before immunization (1–5). Although this background is rather variable, with higher levels observed in individuals in regions of the world with limited sanitation systems, it has been observed in subjects across the World (unpublished data). Yet the nature of these background responses remains largely unknown. Interestingly, it has been recently shown that uninfected individuals can harbor strong immune responses to the *Mycobacterium tuberculosis* (Mtb) bacterium and enteric bacteria such as *Escherichia coli* (*E. coli*) and *Salmonella enterica* serovar Typhimurium (*S*. Typhimurium) (6, 7). These responses are mediated by mucosal associated invariant T (MAIT) cells, a population of T cells that display a TCR Vα7.2^+^CD161^+^ phenotype and which are restricted by the non-classical MHC-related molecule 1 (MR1) (7). After birth, MAIT cells acquire a memory phenotype and expand dramatically, up to 1–4% of human blood T cells (8). Moreover, it has been shown that MAIT cells are abundant in the human intestine, and experiments in mice indicated that their accumulation in the periphery requires B cells and commensal flora (7, 9). Indeed MAIT cells are absent from germ-free mice but can be developed after bacterial colonization (7).

Therefore, it is reasonable to speculate that MAIT cell immune responses to intestinal bacteria might be the source, at least in part, of the background responses observed before immunization. To evaluate this hypothesis we measured MAIT cell immune responses to intestinal microbes, including commensals (i.e., *E. coli* HS and Nissle 1917 strains) and enteric pathogenic bacteria [*S. enterica* serovar Typhi (*S. typhi*), Enteropathogenic *E. coli* (EPEC) and Entero-Invasive *E. coli* (EIEC)] in healthy individuals without a history of enteric bacterial immunization. We found that B cells might be a source of antigen-presenting cells (APCs) to MAIT cells. Indeed, MAIT cells were activated by all bacteria-infected B cells (used as APC in these studies) tested, but not by uninfected cells. These responses were restricted by the non-classical MR1 restricted and involved the endocytic pathway. The quality of these responses (i.e., cytokine profile) was dependent on bacterial load but not on the level expression of MR1 or bacterial antigen on B cell surface, suggesting that a threshold level of MR1 expression is required to trigger MAIT activation. These results provide important insights into the role of B cells as a source of APC to MAIT cells and the gut immune surveillance of commensal microbiota.

## Materials and Methods

### Bacterial strains

Three commensals *E. coli* strains were used, i.e., BL21 [obtained from Dr. Tettelin’s laboratory (laboratory strain derived from a normal commensal of the human gut, isolated from human feces)] (10), HS [obtained from the Center for Vaccine Development (CVD) collection of commensal *E. coli* (clinical isolate)] (11), and *E. coli* Nissle 1917 [kindly provided by Sonnenborn, Ardeypharm, Germany (a probiotic *E. coli* strain)] (12, 13). Three enteropathogens were also used: two *E. coli* strains, i.e., EPEC strain O127H6 [obtained from the CVD collection (reference strain)] and EIEC strain CDC EDL (ATCC, Rockville, MD, USA) and wild type *S. enterica* serovar Typhi (*S. typhi*) strain ISP1820 (obtained from the CVD collection) (1, 2, 5, 14–17). *Listeria monocytogenes* (obtained from the CVD collection) was used as negative control.

### Bacteria media and growth conditions

Luria–Bertani (LB) agar broth Lennox (Difco Laboratories, Detroit, MI, USA) and LB agar Lennox (Difco) were prepared according to the package instructions. For infection experiments with *E. coli* strains, bacteria were grown overnight in LB broth with vigorous shaking (~300 rpm) at 37°C. The following morning, the starter culture was diluted 1/50–1/100 into LB medium, and grown for 2.5–3.0 h. To ensure that the culture did not grow to a high density, measurements the OD600 of the culture were performed every 15–20 min. The cultures were stopped when they approached 0.4, which for most strains of *E. coli* is equivalent to 10^8^ bacteria/ml. The cultures were then pelleted, resuspended in RPMI media (without antibiotics) and used to infect cells. For infection experiments with *S. typhi*, cells were grown as above described and the cultures were stopped when they approached 0.2 which for *S. typhi* is equivalent to 10^8^ bacteria/ml.

*Listeria monocytogenes* were cultured on blood agar plates (5% bovine blood in blood agar base) at 37°C as previously described (18).

### Subjects

Seven healthy volunteers, between 24 and 41 years old, recruited from the Baltimore-Washington area participated in this study. Volunteers were screened for previous vaccination history, good health by medical history, physical examination, and normal laboratory tests, including blood counts, and the absence of antibiotic treatment. Volunteers were explained the purpose of this study and gave informed, signed consent. Peripheral blood mononuclear cells (PBMC) were isolated by density gradient centrifugation and cryopreserved in liquid N_2_. These PBMC were used *ex vivo* as effectors cells. The human experimentation guidelines of the US Department of Health and Human Services and those of the University of Maryland, Baltimore, were followed in the conduct of the present clinical research. All blood specimens were collected from volunteers that participated in the University of Maryland Institutional Review Board approved protocol number HP-00040025 that authorized the collection of blood specimens for the studies included in this manuscript.

### Antibodies, cell culture media, and reagents

Cells were stained with monoclonal antibodies (mAbs) to CD3 (clone UCHT1), CD69 (clone TPI-55-3) (Beckman-Coulter, Miami, FL, USA), CD8 (clone HIT8a), CD161 (clone DX12), interferon (IFN)-γ (clone B27), tumor necrosis factor (TNF)-α (clone MAb11) (BD Pharmingen, San Diego, CA, USA), CD14 (clone TuK4), CD19 (clone SJ25-C1), CD45 (clone H130) (Invitrogen, Carlsbad, CA, USA), interleukin (IL)-17A (clone eBio64DEC17) (eBioscience, San Diego, CA, USA), TCRα7.2 (clone 3C10) (Biolegend, San Diego, CA, USA). These antibodies were directly conjugated to the following fluorochromes: fluorescein isothiocyanate (FITC), Phycoerythrin (PE), PE-Cy7, Energy Coupled Dye or PE-Texas-Red conjugate (ECD), Pacific Blue, Pacific Orange, Alexa 647, allophycocyanin (APC)-Alexa 700, and APC-Cy7.

Anti-MR1 (clone 26.5) (kindly provided by Dr. Ted H. Hansen) as well as anti-MR1 (goat polyclonal) (Santa Cruz Biotechnology, San Diego, CA, USA) and anti-MR1 (rabbit polyclonal) (GeneTex, Irvine, CA, USA) antibodies were used in this manuscript. Unless stated otherwise, surface staining for MR1 proteins were done mainly using anti-MR1 polyclonal antibodies. Of note, all three anti-MR1 antibodies were able to detect MR1-expressing cells at similar levels (Figure S1 in Supplementary Material). However, in our hands MR1 clone 26.5 possessed the best blocking properties and it was chosen as the election antibody for the blocking experiments. Secondary antibodies included FITC-donkey anti-goat IgG (Santa Cruz) and -PE F(ab′)2-Donkey anti-Rabbit IgG (BD Pharmingen). Both secondary antibodies were pre-adsorbed by the manufacturers. Of note, anti-MR1 (GeneTex) antibodies directly conjugated by our group to APC were also used in these studies.

Culture medium consisted of RPMI 1640 (Gibco, Grand Island, NY, USA) supplemented with 100 U/ml penicillin, 100 μg/ml streptomycin, 50 μg/ml gentamicin, 2 mM l-glutamine, 2.5 mM sodium pyruvate, 10 mM HEPES buffer, and 10% heat-inactivated fetal bovine serum (R10).

Purified Lipid A (LPA) (the biologically active component of lipopolysaccharide) from *E. coli* F583 Rd mutant, lactacystin (LC), and cytochalasin D (CCD) were purchased from Sigma) (St. Louis, MO, USA).

### Target cells

Autologous Epstein–Barr virus (EBV)-transformed lymphoblastoid B cell lines (B-LCL), primary B cells, and an epithelial cell line were used as targets. B-LCLs were established from PBMC, described in the above Section “Subjects,” following standard procedures using B95-8 cell line (ATCC) supernatants as the source of EBV (19, 20). After transformation, B-LCL were maintained in culture in R10 medium or cryopreserved until used in the experiments.

Primary B cells were isolated from PBMC by negative selection using untouched human B cell immunomagnetic bead kit (Invitrogen). Isolation was performed as per manufacturer’s instructions. B cell populations were >85% pure as determined by flow cytometric analysis. B cells were used immediately after isolation.

The epithelial cell line used was the human enterocyte cell line (HCT-8) that was originally derived from the junction of the small and large bowels (21). HCT-8 cells were cultured as previous described (17) in R10 medium.

### Infection of target/stimulator cells

Cells were infected as previously described (1, 2, 5, 14–16) with slight modifications. Briefly, target cells were infected by incubation in RPMI (without antibiotics) at 37°C for 2 or 3 h with any of the *E. coli* strains or *S. typhi*, respectively at different multiplicity of infection (MOI). After incubation, cells were washed and incubated for an additional 16–18 h in complete R10 containing gentamicin (100 μg/ml) to kill extracellular and/or to detach cell-bound bacteria. The presence of *E. coli* surface antigens was measured by flow cytometry. To confirm that targets were infected with *E. coli*, cells were stained with protein-A purified anti-*E. coli*-FITC IgG (AbCam). To confirm that targets were infected with *S. typhi*, cells were stained with anti-CSA-1-FITC (KPL, Gaithersburg, MD, USA). In some experiments, targets were pretreated with inhibitors before infection. For pretreatments, cells were incubated with the indicated concentrations of LC and CCD 2 h before infection. Unless stated otherwise, targets were infected at 1:30 MOI.

For co-culture of target and MAIT cells, after treatment with gentamicin targets were irradiated (6,000 rads) and surface stained with anti-CD45, a marker abundantly expressed on the surface of hematopoietic cells (22). This enabled the electronic gating of the target cells during flow cytometric analysis.

### Bacteria entry assays

Bacteria entry experiments were performed as previously described (23–25). Briefly, after gentamicin treatment targets were lysed with 1% Triton X-100 in PBS, diluted to 1 ml with LB broth, and 100 μl aliquots were plated on LB agar. Colonies were counted after an overnight incubation.

### Surface and intracellular staining

CD45-stained-target cells were co-cultured with overnight-rested *ex vivo* PBMC at a PBMC to target cell ratio of 5:1. PBMC cultured with uninfected target cells or *Staphylococcus* enterotoxin B (SEB) (10 μg/ml, Sigma, St. Louis, MO, USA) were used as negative and positive controls, respectively. After 1–2 h of stimulation, protein transport blockers, Monensin (1 μg/ml, Sigma) and brefeldin-A (BFA) (2 μg/ml, Sigma), were added to the co-culture. After overnight (16–18 h) incubation, cells were harvested, stained with a dead-cell discriminator, violet fluorescent viability dye (ViViD, Invitrogen) (26), followed by surface staining with mAbs against surface antigens (CD3, CD8, CD14, CD19, TCRα 7.2, and CD161) and fixation and permeabilization with Fix and Perm cell buffers (Invitrogen, Carlsbad, CA, USA). Cells were then stained intracellularly for IFN-γ, TNF-α, IL-17A, and CD69. Finally, cells were fixed and analyzed by flow cytometry on an LSR-II instrument (BD Biosciences). Data were analyzed with WinList v7.0 (Verity Software House, Topsham, ME, USA). Lymphocytes were gated based on their scatter characteristics. Single lymphocytes were gated based on forward scatter height vs. forward scatter area. A “dump” channel was used to eliminate dead cells (Violet Viability Dye; ViViD^+^) as well as macrophages/monocytes (CD14^+^), B lymphocytes (CD19^+^), and targets (CD45^+^) from analysis. This was followed by additional gating on CD3, CD8, TCR Vα7.2, CD161 to identify cytokine-producing (IFN-γ, TNF-α, and IL-17A) MAIT cells.

#### Statistical analysis

All statistical tests were performed using Prism software (version 5.02, GraphPad software, La Jolla, CA, USA). Comparisons between groups were performed using Pearson Product Moment Correlation tests. *p* Values <0.05 were considered significant.

## Results

### Expression of *E. coli* antigens on target cells

To investigate the role of MAIT cells in detecting and responding to commensal bacteria, we decided to modify an experimental protocol previously described for *S. typhi* infection (1, 5, 14, 16, 17) to enable cellular infection and antigen presentation of commensal bacteria antigens. We used *E. coli* and lymphoblastoid B cell line (B-LCL) transformed by EBV as models of commensal bacteria and APC, respectively. This strategy was based on three main reasons. First, *E. coli* are normal residents of the gastrointestinal tract. They are traditionally extracellular organisms that in certain conditions can result in opportunistic intracellular infections (27). Second, B cells are believed to be main contributors as APC during the initial phases of host immune responses (28), and are required for MAIT cell accumulation in the periphery (9). Third, our group has a long track record of using B-LCL cells as APC, in particular for bacterial antigen presentation (1, 2, 5, 16). Indeed, these cells have demonstrated to be a very attractive model because of their easiness to culture and their robustness to survive after bacterial infection. Moreover, B cells are known to express the CD161 MAIT cell ligand Lectin-like transcript-1 (LLT1) (29–31).

**Figure 1:**
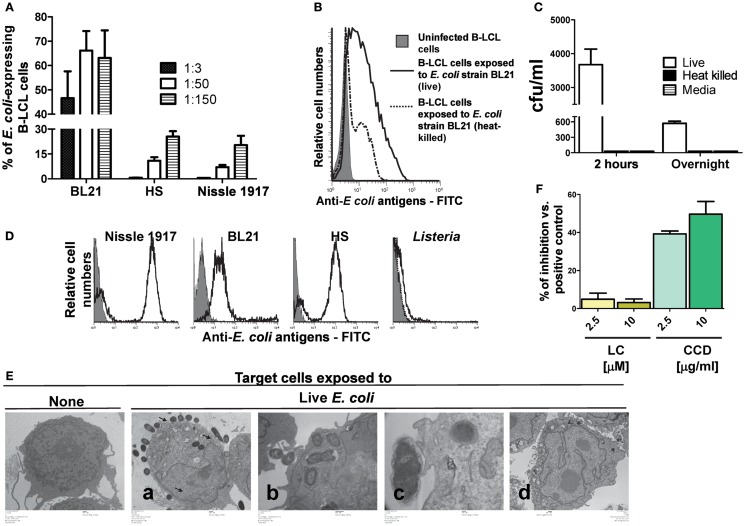
**Expression of *E. coli* antigens on B-LCL target cells**. B-LCL cells were infected with *E. coli* for 2 h followed by gentamicin treatment. **(A)** B-LCL cells were infected with *E. coli* at different multiplicity of infection (MOI, 1:3, 1:50, and 1:150) by either of the three strains: BL21, HS, or Nissle 1917 for 2 h. The percentage of the *E. coli*-expressing cells was assessed by flow cytometry after overnight treatment with gentamicin. Bars represent mean ± SE. **(B)** B-LCL cells were also exposed to live or heat-killed *E. coli* strain BL21 and the percentage of *E. coli*-expressing cells was evaluated by flow cytometry after overnight treatment with gentamicin or **(C)** by counting the number of colony-forming units (CFU) in cell lysates upon culture on agar plates. B-LCLs were infected for 2 h followed by 2 h or overnight treatment with gentamicin. Media (without B-LCL cells) was used as control for the presence of extracellular bacteria. The CFU was normalized per milliliter. **(D)** To confirm that anti-*E. coli* antibodies detect all *E. coli* strains being studied, these *E. coli* strains as well as *Listeria* (negative control) were directly stained with *E. coli* antibodies and analyzed by flow cytometry. **(E)** Infection was further confirmed by electron microscopy. B-LCL cells were left untreated (none, lower magnification) or exposed to live *E. coli* strain BL21 at a MOI of 1:100 [from left to right, lower **(a)**, medium **(b)**, or higher **(c)** magnification] and treated with gentamicin for 2 h. After 2 h, B-LCL cells were washed and cultured overnight in complete media with gentamicin **(d)** to control for bacteria detachment. **(F)** In some experiments B-LCLs were pretreated with lactacystin (LC) and cytochalasin D (CCD) for 2 h at different concentrations before infection. Data are representative of five **(A)**, three **(B)**, and two **(C–F)** experiments.

Herein, as a preliminary step, we evaluated whether the non-pathogenic *E. coli* strains BL21, HS, and Nissle 1917 were able to infect B-LCL targets and express antigens on their cell membranes. To this end, B-LCLs were incubated with medium alone or with any of the three *E. coli* strains for 2 h at different MOI. After incubation, cells were washed and incubated for an additional 16–18 h in complete RPMI containing gentamicin (100 μg/ml) to kill extracellular and/or to detach cell-bound bacteria. The presence of *E. coli* surface antigens was measured by flow cytometry. We observed that while all selected strains were able to express *E. coli* antigens on the B-LCL cell membrane, they did it at different levels. The percentage of cells expressing *E. coli* antigens was much lower on B-LCLs exposed to HS and Nissle 1917 strains as compared with those cells exposed to the BL21 strain (Figure 1A). In contrast, no significant expression of *E. coli* antigens was observed on the cell membrane of uninfected cells (Figure 1B). We also observed that cells exposed to heat-killed *E. coli* had substantially lower levels of *E. coli* antigens on the cell surface as compared to cells exposed to live *E. coli* (Figure 1B). These results suggest that expression of *E. coli* antigens on the cell membrane is largely dependent on bacterial cell invasion.

To validate these findings, we next investigated the presence of viable intracellular bacteria. In these experiments, *E. coli* strain BL21, which induced high levels of expression of *E. coli* antigens on the B-LCL cell membrane, was chosen as a model for bacterial cell invasion. As described above, B-LCL were co-cultured with *E. coli* for 2 h. After incubation, cells were treated with gentamicin for an additional 2 or 16–18 h (overnight) to eliminate extracellular bacteria. Cells were then extensively washed and lysed with 1% Triton X-100. An aliquot of cell lysate was plated on LB agar and BL21 colonies were counted after an overnight incubation. Media with bacteria cultured alone (i.e., without B-LCL cells) were used as control. As expected, cells exposed to heat-killed bacteria or media only (no cells) demonstrated virtually no bacteria growth on LB agar. In contrast, easily detectable colony-forming units (CFU) were present in cells exposed to live bacteria (Figure 1C). To confirm that anti-*E. coli* antibodies bind to all *E. coli* strains being studied, the three strains of *E. coli*, as well as a strain of *Listeria* (negative control) were directly stained with *E. coli* antibodies and analyzed by flow cytometry. We observed that while the median fluorescence intensity (MFI) was different for each of the three *E. coli* strains (Nissle 1917 > HS > BL21), all of them were detected by anti-*E. coli* antibodies (Figure 1D; Figure S2 in Supplementary Material). These findings prompted us to examine the antibody binding to *E. coli* by flow cytometry after adding different dilutions of the purified polyclonal antibody preparation to the three *E. coli* strains followed by the addition of anti-rabbit PE. As shown in Figure S2 in Supplementary Material, the MFI revealed that higher antibody concentrations were required to saturate Nissle 1917 and HS strains than BL21, suggesting that the latter have less biding antigens available. The specificity of this polyclonal antibody preparation was confirmed by the low/minimal binding observed with *L. monocytogenes* (Figure 1D). Electron microscopy examination of the infected B-LCL cells confirmed the presence of intracellular *E. coli* BL21 strain. As shown in Figure 1E; 2 h after infection, intracellular bacteria could be seen in the cytoplasm of B-LCL (Figure 1Ea, second panel from left to right). We also observed bacteria in close proximity to the cells with the cell membrane beginning to extend around individual bacteria (Figure 1Eb, third panel from left to right). Finally, some bacteria were seen to induce pedestal-like formation, a critical step during *E. coli* colonization (32) (Figure 1Ec, fourth panel from left to right). As previously reported (5, 16, 33), no surface-bound bacteria were observed after an overnight treatment with gentamicin (Figure 1Ed, last panel from left to right). Taken together, these results demonstrated the ability of *E. coli* to efficiently infect a large percentage of B-LCL. Based on these observations we hypothesized that the presence of live bacteria inside of the cells might increase the likelihood that *E. coli* proteins are being processed and gain access to the host cell MHC class I antigen processing pathways required for CD8^+^ T cell recognition. To test this assumption, we examined the effect of LC, an inhibitor of proteasome processing (14, 34), and the actin-depolymerizing agent CCD (35, 36), an inhibitor of endocytosis, on *E. coli* antigen expression on B-LCLs using flow cytometric assays. B-LCLs were pretreated with LC or CCD for 2 h at different concentrations before infection. Pretreatment of the cells with LC did not lead to a decrease in the percentage of *E. coli*-expressing B-LCLs. Instead, we observed an important decrease in the percentage of *E. coli*-expressing B-LCLs in the presence of CCD (Figure 1F). Together, these data suggested that *E. coli* antigen expression on targets is a result of both endocytosis and bacterial invasion.

### Infection-induced MR1 expression

**Figure 2:**
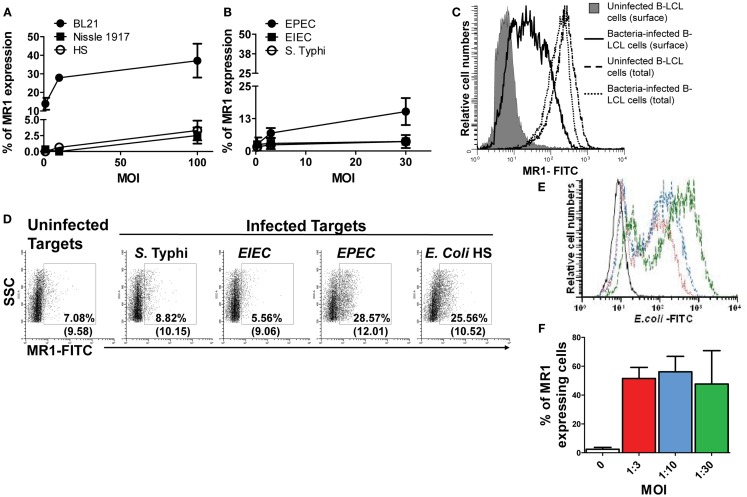
**MHC-related molecule 1 proteins are exported to the surface of bacteria-infected-cells**. B-LCL cells were infected with either commensals **(A)** (i.e., *E. coli* strains BL21, Nissle 1917, or HS) or pathogenic bacteria **(B)** (i.e., *EPEC* or *EIEC* or *S. typhi*) for 2 h at different multiplicity of infection (MOI) (1:1, 1:10, and 1:100 for commensals and 1:0.3, 1:3, and 1:30 for pathogenic strains). Cells left uninfected were used as controls. After 16–18 h of gentamicin treatment cells were stained with MR1 antibodies and analyzed by flow cytometry. **(C)** Permeabilized (total) and non-permeabilized (surface) B-LCL targets were stained with anti-MR1 antibodies. Percentages of MR proteins on uninfected (solid histogram and dashed line) and BL21-infected-cells (MOI 1:30) (full and dotted lines) were determined by flow cytometry. **(D)** HCT-8 epithelial cells were left uninfected or infected with either *S. typhi, EIEC, EPEC*, or *E. coli* HS at an MOI of 1:100 for 2 h. After 16–18 h of gentamicin treatment cells were stained with MR1 antibodies and analyzed by flow cytometry. Numbers correspond to the % of MR1 positive cells followed by mean fluorescence intensity (MFI) of positive cells (in parenthesis). Primary B cells were also infected with *E. coli* strain BL21 at different MOI (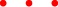 , 1:3; 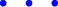 ,1:10; and 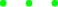 , 1:30) and surface expression of *E. coli* antigens **(E)** and MR1 expression **(F)** measured by flow cytometry. Data are representative of five **(A,B)** and two **(B–F)** experiments.

Although, it has been demonstrated that human MAIT cells recognized MR1 expressed on infected targets over-expressing human MR1 (7), the MR1 surface expression of endogenous MR1 on target cells following bacterial infection has not yet been definitively shown. To our knowledge, up-regulation of MR1 has been limited to a modest increase in the A549 epithelial cell line (6). Thus, we next investigated MR1 expression on the cell surface of B-LCL cells *in vitro* infected with various bacteria. As described above, B-LCL were cultured with medium only or with *S. typhi* or any of five *E. coli* strains: commensals (i.e., *E. coli* strains BL21, Nissle 1917, or HS) or pathogenic bacteria (i.e., EPEC and EIEC). B-LCLs were then extensively washed and treated with gentamicin to eliminate extracellular bacteria. After 16–18 h, B-LCL cells were stained with MR1 antibodies and analyzed by flow cytometry. While B-LCL cells infected with BL21 and *EPEC* induced high levels of MR1 on the cell surface, B-LCL cells infected with *E. coli* strains HS and Nissle 1917, *EIEC*, and *S. typhi* induced only low levels of MR1 on the cell surface (Figures 2A,B). Because previous work has shown the presence of MR1 proteins intracellularly in different types of B and T cell lines (37), we speculated that MR1 proteins might have been exported to the cell surface from a pool of endogenous MR1 already present in the cytoplasm of target cells. Thus, B-LCL targets were either surface stained only (non-permeabilized cells) or surface and intracellular stained (total; permeabilized cells) with anti-MR1 antibodies. Percentages of MR1 proteins expressed on uninfected and BL21-infected B-LCL cells (MOI 1:30) were determined by flow cytometry. While MR1 expression on the surface of the target cell was restricted to targets infected with BL21 strain, expression of total MR1 was found to be similar on both uninfected and infected targets (Figure 2C). These results support our hypothesis that bacterial infection might provide a signal to endogenous MR1 proteins to be expressed at the cell surface.

We next investigated whether the increase in MR1 expression occurs in a more physiological situation. Because following ingestion *E. coli* and *Salmonella* are far more likely to first encounter epithelial cells and then B cells, we evaluated surface MR1 expression on HCT-8 epithelial cells. These epithelial cells are human enterocyte cell lines that were originally derived from the junction of the small and large bowel (21). The choice of this cell line was based on our previous work demonstrating its ability to secrete cytokines (e.g., IL-1, IL-6, IL-8, IL-11, IL-12p70, IL-17A, IL-21, and TNF-α following exposure to *S. typhi* (17). In agreement with the above results using B-LCL cells, MR1 expression was increased on HCT-8 cells after bacterial infection (Figure 2D). Like for B-LCL cells the increases differed depending on the bacterial strain tested. Interestingly, increases in MR1 expression were observed on HCT-8 cells infected with both *EPEC* and *E. coli* strain HS but not with *S. typhi* or *EIEC* (Figure 2D). To further confirm our findings with B-LCL cells, we deemed of great importance to investigate the effects of bacteria on MR1 expression on primary human B cells. B cells purified from PBMCs were infected with *E. coli* strain BL21 at different MOI and MR1 expression measured by flow cytometry. As for B-LCLs, a high proportion of *E. coli*-infected primary B cells expressed MR1 proteins on their cell membrane, compared to minimal MR1 expression in uninfected cells (Figures 2E,F).

**Figure 3:**
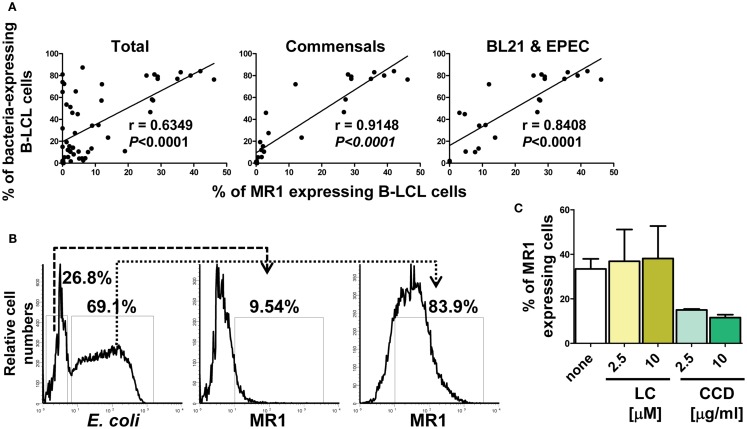
**Correlation between MR1 up-regulation and levels of bacterial infection**. **(A)** Percentage of both MR1 and bacterial antigens on the surface of B-LCL cells were measured by flow cytometry. Results were divided into three groups: (1) total, B-LCL cells infected with any bacteria tested (commensals and pathogenic bacteria), (2) B-LCL cells infected with commensals only, and (3) B-LCL cells infected with bacteria that induce high levels of MR1 on the cell surface (BL21 and *EPEC*). Correlation coefficients “*r*” and “*p*” values are shown. *p* Values of <0.05 were considered statistically significant. Comparisons between groups were performed using Pearson Product Moment Correlation tests. **(B)** A representative flow cytometric experiment using polyclonal antibodies to *E. coli* antigens and to MR1. BL21-infected B-LCL were gated based on forward and side scatter (FS/SS) and then through a “dump” channel used to eliminate dead cells (ViViD^+^). B-LCL were then gated as *E. coli*^−^ and *E. coli*^+^ respectively, and analyzed for their MR1 surface expression. **(C)** MR1 expression in the absence (none) or in the presence of inhibitors lactacystin (LC) and cytochalasin D (CCD) at different concentrations. B-LCLs were pretreated with LC and CCD for 2 h before infection. Data are representative of five **(A)** and two **(B,C)** experiments.

We next investigated whether there is a relationship between MR1 up-regulation and infection levels. To this end, results on the percentages of both MR1 and bacterial antigens expressed on the surface of bacteria-infected B-LCL cells were divided into five groups: (1) total, B-LCL cells infected with any bacteria tested (commensals and pathogenic bacteria), (2) B-LCL cells infected with commensals only (*E. coli* strains BL21, HS, and Nissle 1917), (3) B-LCL cells infected with pathogenic bacteria only (*S. typhi*, EPEC, and EIEC), (4) B-LCL cells infected with bacteria that induce low levels of MR1 on the cell surface (*E. coli* strains HS and Nissle 1917, *EIEC* and *S. typhi*), and (5) B-LCL cells infected with bacteria that induce high levels of MR1 on the cell surface (BL21 and *EPEC*). Comparisons among the different groups were performed using Pearson Product Moment Correlation. We found a striking correlation between MR1 up-regulation and infection in the total group (*p* < 0.0001) (Figure 3A). These correlations were driven by B-LCL cells infected with BL21 and EPEC (*p* < 0.0001) (Figure 3A). No correlations were found in the group with B-LCL cells infected with pathogenic bacteria only or in the group with B-LCL cells infected with bacteria that induce low levels of MR1 expression on the cell surface (*p* values = 0.438 and 0.751, respectively) (data not shown). Finally, simultaneous flow cytometric measurements of MR1 and *E. coli* antigens at the single cell level showed that the vast majority of cells expressing MR1 were also expressing *E. coli* antigens (Figure 3B; Figure S3C in Supplementary Material). To further understand the mechanism of MR1 up-regulation after bacterial infection, we examined the effect of LC and CCD on MR1 expression on E. coli-infected B-LCLs using flow cytometric assays. B-LCLs were pretreated with LC or CCD for 2 h at different concentrations before infection. Similar to *E. coli* antigen expression, pretreatment of the cells with LC did not lead to a decrease in the percentage of MR1-expressing B-LCLs. Instead, we observed an important decrease in the percentage of MR1-expressing B-LCLs in presence of CCD (Figure 3C; Figure S3C in Supplementary Material). Because it remained possible that recognition of bacteria by B cells was sufficient to up-regulate MR1 without bacterial endocytosis, we examined MR1 up-regulation in B-LCL stimulated with Lipid A (LPA), the hydrophobic anchor of lipopolysaccharide (LPS). LPA is known to signal independently of phagocytosis, and therefore independently of CCD. It is also known that the actin cytoskeleton is not only important for phagocytosis but also for cell signaling through several receptors. Specifically, recent studies have shown that under altered physiological conditions LPS might be able to stimulate human B cells (38, 39). Thus, to evaluate this possibility, B-LCL cells were cultured in medium alone or with purified LPA (1, 3, and 10 μg/ml) and MR1 up-regulation evaluated by flow cytometry. No MR1 up-regulation was observed at any of the LPA concentrations evaluated (Figure S3A in Supplementary Material). To control for autofluorescence and background non-specific staining resulting from the rabbit polyclonal anti-MR1 antibodies, we stained uninfected and *E. coli*-infected B-LCL cells with normal rabbit sera (non-immune rabbit). As shown in Figure S3C in Supplementary Material, we observed little or no staining in the cells treated with normal rabbit sera, indicating that polyclonal rabbit anti-MR1 antibodies were specifically associated with MR1 antigen. Moreover, to exclude the possibility that our observations were due to cross-reactivity between antibodies to MR1 and *E. coli* antigens we stained *E. coli* strain BL21 bacteria with the polyclonal rabbit anti-MR1 antibodies. As shown in Figure S3D in Supplementary Material, only marginal cross-reactivity was observed.

These results suggest that bacterial-induced MR1 expression relies on an endocytic event rather than on direct bacterial contact to the cell surface or on a proteasome-dependent pathway. These results are in agreement with previous work using an MR1 over-expressing target system (40). Taken together, these results demonstrated that bacterial infection might trigger MR1 to be expressed at the cell surface using mainly endocytic pathway. Moreover, this up-regulation might be a function of both, the type target cell and the bacteria strain.

### MAIT are activated by bacteria-infected B cells

**Figure 4:**
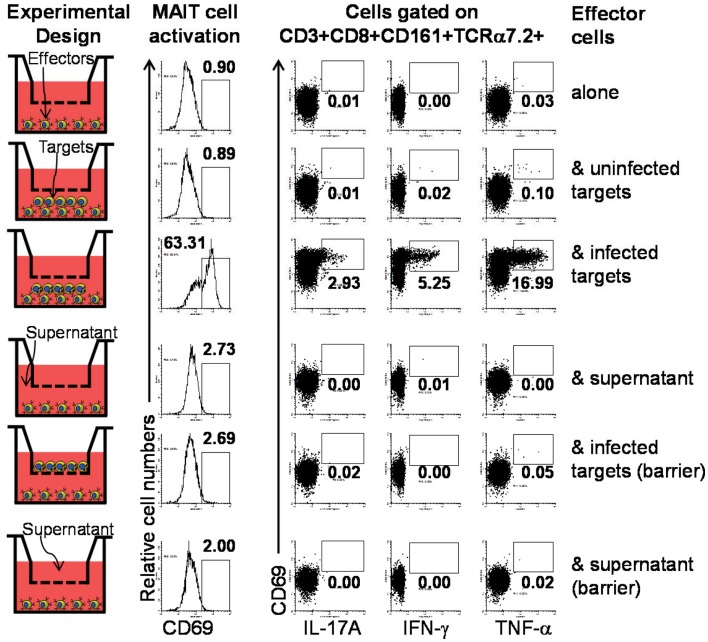
**Requirement of cell-to-cell contact for MAIT cells activation by primary B cells**. We investigated the role of cell-to-cell contact in the MAIT cell activation by performing experiments using transwell filters. In these experiments MAIT cells were cultured in direct contact with target cells (uninfected or BL21-infected B-LCLs) or separated by transwell filters (0.4 μm). Supernatants from infected-target cell culture that were cleared by centrifugation were used as control for microbial product effect. Human *ex vivo* PBMC were exposed for 16–18 h to B-LCL targets and activation and cytokine production by MAIT cells were evaluated by flow cytometry. Data are representative of two experiments.

**Figure 5:**
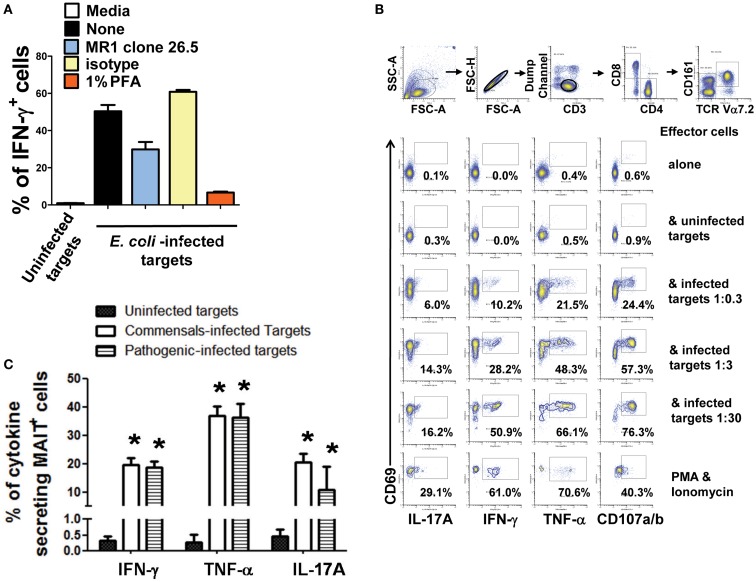
**Cytokine production by MAIT cells stimulated with bacteria-infected-cells**. **(A)** MAIT cells were exposed to either uninfected (media) or BL21-infected B-LCL cells that were treated or not (none) with monoclonal antibodies to MR1 antigens (clone 26.5), isotype control, or fixed in 1% of paraformaldehyde (PFA). After 16–18 h co-culture, IFN-γ^+^ MAIT cell was evaluated by flow cytometry. **(B)** Percentage of MAIT cells secreting either IFN-γ, TNF-α, or IL-17A cytokines when exposed to primary B cells infected with *E. coli* strain BL21 at different MOI (1:0.3, 1:3, and 1:30). Single lymphocytes were gated based on forward scatter height vs. forward scatter area. A “dump” channel was used to eliminate dead cells (ViViD^+^) as well as macrophages/monocytes (CD14^+^), B lymphocytes (CD19^+^), and targets (CD45^+^) from analysis. This was followed by additional gating on CD3, CD8, TCRα7.2, and CD161 to identify CD107a/b^+^ and cytokine-producing (IFN-γ, TNF-α, and IL-17A) MAIT cells. **(C)** Percentage of MAIT cells secreting either IFN-γ, TNF-α, or IL-17A cytokines when exposed to commensal (i.e., *E. coli* BL21, HS, or Nissle 1917 strains) or enteric pathogenic [*S. typhi*, Enteropathogenic *E. coli* (EPEC), or Entero-Invasive *E. coli* (EIEC)] bacteria-infected B-LCL cells were also evaluated. Bars represent mean ± SE. *p* Values of <0.05 were considered statistically significant. *Uninfected vs. either commensal or pathogenic-infected targets. Comparisons between groups were performed using Kruskal–Wallis One Way Analysis of Variance on Ranks. Data are representative of two **(A,B)** and five **(C)** experiments.

Despite of the reported B cell requirement for MAIT cell expansion in the periphery (8), direct activation of MAIT cells by B cells has not yet been reported (7). To directly evaluate this phenomenon, we investigated the role of cell-to-cell contact in MAIT cell activation by performing experiments using transwell filters. In these experiments MAIT cells were cultured in direct contact with target cells (uninfected or BL21-infected B-LCLs) or separated by transwell filters (0.4 μm). Supernatants from infected-target cultures that were cleared by centrifugation were used as controls for microbial product effect. Human *ex vivo* PBMC were exposed for 16–18 h to B-LCL targets and activation and cytokine production by MAIT cells were evaluated by flow cytometry. We selected 16–18 h to minimize bystander stimulation while maintaining a close parallel with the *in vivo* cytokine response (41). CD69, a very early activation marker on lymphocytes, was used to monitor MAIT cell activation (42). MAIT cells were identified as the double positive TCR Vα7.2^+^ CD161^+^ population. Under these experimental conditions, MAIT cells in contact with infected B-LCLs, had an increase in both the percentage of the early activation marker CD69 and production of IL-17A IFN-γ and TNF-α cytokines (Figure 4). In contrast, marginal or no increases were observed in the percentage of activated or cytokines expressing MAIT cells when cultured alone, in contact with uninfected B-LCLs, in the presence of supernatant or separated from infected B-LCLs by transwell filters. These results suggest that the increase in the percentage of activated and cytokine-secreting MAIT cells was dependent on cell-to-cell interactions (e.g., TCR engagement). These results also demonstrated that soluble factors such microbial products are not essential for MAIT cell activation in the absence of target cells. To confirm the specificity of the MAIT cell activation, MAIT cells were exposed to *Listeria*-infected targets which has previously been shown not to be activated by *L. monocytogenes* (43). As expected MAIT cells only marginally responded to targets infected with *Listeria* as compared to *E. coli* strain BL21-infected targets. These results further confirmed the specificity of MAIT cell responses to members of the *Enterobacteriaceae* family, which includes *E. coli* and *Salmonella* (Figure S4 in Supplementary Material). Moreover, the frequency of IFN-γ^+^ MAIT cells was markedly reduced when infected-target cells were treated with mAbs to MR1 (clone 26.5) (Figure 5A). In these experiments, B-LCL cells were infected with *E. coli* strain BL21, treated or not (none) with mAbs to MR1 antigens (clone 26.5, 10 μg/ml) or IgG2 isotype control for 2 h and then exposed to MAIT cells. Because previous work has shown the ability of fixed targets to activated MAIT cells (6, 7, 44), we also compare frequency of IFN-γ^+^ MAIT cells exposed to live infected targets or infected targets fixed in 1% paraformaldehyde. Although infected targets fixed in 1% paraformaldehyde were able to trigger an increase in the levels of IFN-γ^+^MAIT cells as compared to controls (uninfected targets), levels of IFN-γ^+^ MAIT cells exposed to fixed targets were substantially lower than MAIT cells exposed to live infected targets (Figure 5A). These results support the contention that bacteria-infected targets expressing MR1 on the cell surface are involved in MAIT cell activation. Moreover, based on the results of the experiments using fixed targets, we could also propose that MR1 in a particular conformation on the cell surface might be needed for an optimal MAIT cell activation.

Although, B-LCL have intrinsic differences when compared to primary B cells, it has been demonstrated that B cells display the same set of antigens observed in B cell lines (45). To test this assumption, MAIT cells were exposed to primary B cells isolated by negative selection from PBMC of healthy volunteers and infected with *E. coli* strain BL21 at different MOI (1:0.3, 1:3, and 1:30) in the presence of mAbs to CD107 a and b. The CD107 a and b antibodies were used to measure degranulation (46). MAIT cells alone/or in the presence of uninfected targets or phorbol myristate acetate (PMA, 50 ng/ml) plus ionomycin (1 μg/ml) were used as negative and positive controls respectively. As shown in Figure 5B, MAIT cells were stimulated in a dose dependent fashion by primary B cells infected with *E. coli* strain BL21. MAIT cells were able to secrete IL-17A, IFN-γ, TNF-α, and express CD107a/b. Taken together, these results are consistent with the hypothesis that B cells are able to present antigens to MAIT cells.

We next evaluated cytokine production by MAIT cells exposed to B-LCL cells infected individually with each of the commensals (i.e., *E. coli* strains BL21, Nissle 1917, or HS) or enteric pathogenic (i.e., EPEC and EIEC) bacteria. We found that MAIT cells were able to secrete IFN-γ, IL-17a, and TNF-α cytokines when exposed to B-LCL cells infected with any of the commensal or enteric pathogenic bacteria strains evaluated. Moreover, we observed that the levels of cytokine production were indistinguishable between MAIT cells stimulated by commensals or by pathogenic bacteria. Aggregate data is shown in Figure 5C. Of note uninfected B-LCLs triggered only limited cytokine production by MAIT cells (Figure 5C). These results are in agreement with those obtained by other groups using different MAIT cell stimulation systems showing the ability of MAIT cells to secrete IFN-γ, IL-17a, and TNF-α cytokines (9, 47). These results also support previous studies suggesting that the MAIT TCR acts like a pattern recognition receptor, with a conserved MR1-binding mode, irrespective of the source of bacterial stimulation (48).

**Figure 6:**
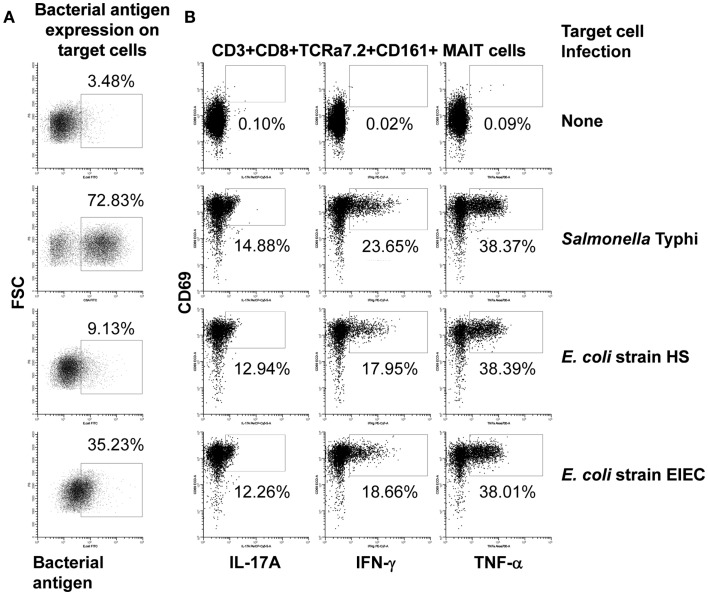
**Bacterial antigen expression and secretion of cytokines by MAIT cells**. B-LCL cells were left uninfected (none) or infected with either *Salmonella typhi* or *E. coli* strains HS or EIEC. **(A)** Percentage of B-LCL cells expressing bacterial antigens on their surface were measured by flow cytometry. Targets infected with *E. coli* or *S. typhi* were stained with antibodies to *E. coli* or CSA, respectively. **(B)** Percentage of cytokine-secreting MAIT cells after 16–18 h of co-culture with uninfected or infected-B-LCL targets. Cytokine production by MAIT cells was evaluated by flow cytometry. Data are representative of five experiments.

We next investigated to what extent the proportion of MAIT cells secreting cytokines is influenced by the level of expression of MR1 or bacterial antigen expression on bacteria-infected targets. Interestingly, although we found a correlation between MR1 and bacterial antigen expression on bacteria-infected B-LCL targets, there were no significant correlations between changes in the expression of MR1 or bacterial antigen expression on bacteria-infected targets and the proportion of MAIT cells secreting cytokines (data not shown). As shown in Figure 6A; B-LCL targets infected by different bacteria (i.e., *S. typhi* and *E. coli* strains HS or EIEC) at the same MOI (1:30) induced different levels of infection on the target cells but similar levels of MAIT cells secreting cytokines (Figure 6B). In addition, dose-response studies showed that expression of bacterial antigens on the cell surface depends on the MOI of bacteria being added. However, no direct correlations were observed between the levels of expression of bacteria on the cell surface of APC and MAIT cell activation (Figure S5 in Supplementary Material). These results suggest that MAIT cell responses might be independent of the levels of MR1 or bacterial antigen expression on bacteria-infected targets, perhaps after a certain expression threshold is achieved.

### MAIT and multi-functional cytokine production

**Figure 7:**
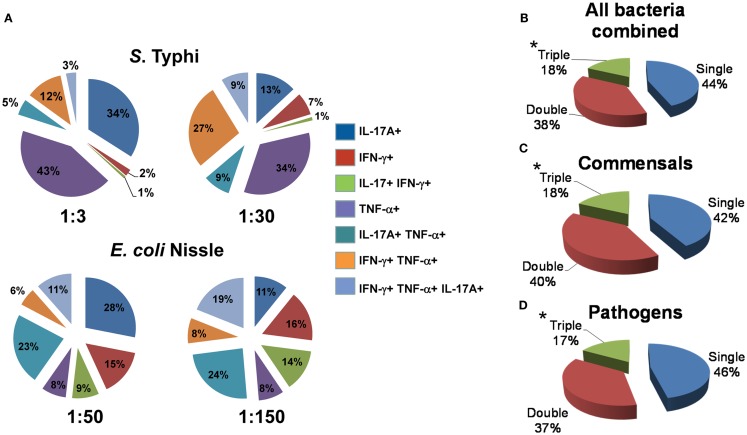
**Multi-functional response of MAIT cells against commensal and pathogenic bacteria**. **(A)** Immune responses of MAIT cells against commensal *E. coli* strain Nissle (MOI 1:50 and 1:150) and pathogenic *Salmonella typhi* (MOI 1:3 and 1:30) are shown. The response patterns are color-coded by MAIT cells producing the indicated cytokines. Numbers represent the percentages of MAIT cells secreting each cytokine combination. **(B–D)** Pie charts show the proportion of MAIT cells producing 1, 2, or 3 cytokines in response to stimulation with bacteria-infected targets. Target cells were infected individually with commensal (*E. coli* strains BL21, HS, or Nissle 1917) or pathogenic bacteria (*S. typhi*, EPEC, or EIEC). Regardless of the type of stimulator cells, statistical differences were found only between MAIT cells secreting 1 (single) or 2 (double) vs. 3 (triple) cytokines. *p Values of <0.05 were considered statistically significant. No statistical differences were found among the three pie charts: **(B)** MAITs exposed to any bacteria-infected-cells (i.e., commensal or pathogenic), **(C)** MAITs exposed to cells infected with commensal bacteria, and **(D)** MAITs exposed to cells infected with pathogenic bacteria. Comparisons between groups were performed using Kruskal–Wallis One Way Analysis of Variance on Ranks. Data are representative of five experiments.

Because our previous work supports the idea that multi-functional T cells might contribute to effective *Salmonella* immunity (5, 49), we then investigated the cytokine secretion patterns of MAIT cells after exposure to infected B-LCL target cells. We measured simultaneously three MAIT cell functions (IFN-γ, TNF-α, and IL-17A cytokine secretion) by multichromatic flow cytometry using the FCOM feature of WinList software which provides the % of cells expressing each of the seven possible cytokine combinations. Analyses of multiple cytokine patterns revealed that the majority of MAIT cell responses were characterized by single or double cytokine producers (Figure 7). Interestingly, at low bacterial loads, IL-17A production in combination with other cytokines was mostly linked to the presence of TNF-α. In most of the individuals studied, at low bacterial loads of *E. coli*, MAIT cells that produce concomitantly IFN-γ and IL-17A but not TNF-α were present a very low frequency or absent but their frequency increased proportionally to the bacterial load. We also observed that the levels of MAIT cells that produce IL-17A were markedly lower in those stimulated by B-LCL targets exposed to high bacteria loads than those MAIT cells stimulated by B-LCL targets exposed to low bacteria loads. Surprisingly, no significant differences were observed between cytokine patterns of MAIT cells stimulated by either commensals or pathogens (Figures 7B–D). Furthermore, regardless of the type of bacteria used to infect B-LCL cells, a statistically significant difference was observed among triple vs. single and double positive MAIT cells (Figures 7B–D). In fact the majority of MAIT cells were single or double producers of cytokines. Interestingly, the levels of MAIT cells that produce triple cytokines were markedly higher in those stimulated by B-LCL targets exposed to high bacteria loads than those of MAIT cells stimulated by B-LCL targets exposed to low bacteria loads. Thus, it appears that the quality of MAIT cell responses was dependent on bacterial load rather than whether the bacteria is commensal or pathogenic.

**Figure 8:**
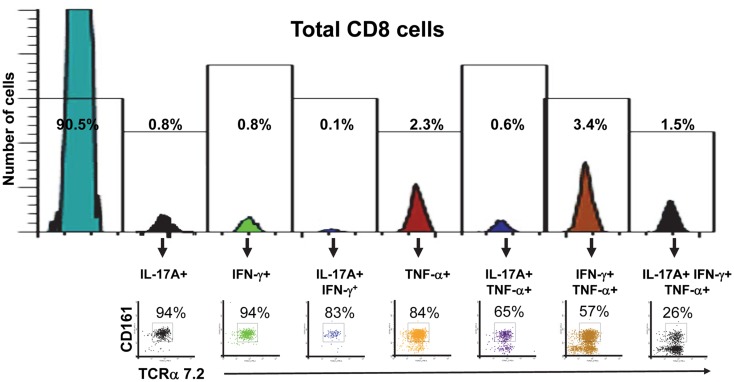
**Proportion of mono-functional and multi-functional MAIT cells among CD8^+^ cells**. FCOM, an analysis tool which automatically reduces multiparameter data to a series of multiple event acquisition gates, one for every possible sub-phenotype, was employed to study CD8^+^ cell multifunctionality. Based on the defined positive staining regions, FCOM calculated seven possible phenotypes, and displayed the event frequency (%) for each of the seven sub-phenotypes in the separate gates shown in the figure. Single lymphocytes were gated-out based on forward scatter height vs. forward scatter area. A “dump” channel was used to eliminate dead cells (ViViD^+^) as well as macrophages/monocytes (CD14^+^), B lymphocytes (CD19^+^), and targets (CD45^+^) from analysis. This was followed by additional gating on CD3 and CD8 to identify multi-functional CD8^+^ cells. Subsequent gates on TCRα7.2 and CD161 were used to identify MAIT cells among each of the seven defined sub-phenotypes of CD8^+^ cells. The data is representative of one experiment showing mono-functional or multi-functional MAIT cell responses to B-LCL cells infected with *E. coli* strain HS at a multiplicity of infection (MOI) of 1:200. Data are representative of five experiments.

Finally, we investigated the proportion of mono-functional and multi-functional MAIT cells among CD8^+^ T cells. We found that the proportion of MAIT cells among CD8^+^ T cells decreased as these CD8^+^ T cells became more multi-functional (Figure 8). We also observed that the majority of mono-functional CD8^+^ T cells were MAIT cells (Figure 8). These results reinforce the innate character of the MAIT cell immune responses bridging the adaptive T cell immune responses.

## Discussion

These studies were directed to understand the origins of the background T cell immune responses observed before immunization in individuals who are candidates to receive enteric bacterial vaccines (1–5). A prevailing hypothesis is that these background responses were due to the presence of cross-reactive T cells acquired during previous infections by others enteric pathogens. A shortcoming of this hypothesis is the fact that varying levels of these background responses have been observed in most individuals regardless of a history of previous or current enteric infections.

Recently it has been shown that uninfected individuals can harbor strong immune responses to Mtb as well as to enteric bacteria such as *E. coli* and *Salmonella* (6) and that these responses are mediated by MAIT cells. MAIT cells are a population of T cells that display a TCR Vα7.2^+^ CD161^+^ phenotype and are restricted by the non-classical MR1 molecule (7). Of note, MAIT cells are abundant in the human intestine, and studies in mice suggest that their accumulation in the periphery requires B cells and commensal flora (7, 9). Therefore, we speculated that MAIT cell immune responses directed not only to pathogens but also against commensals might explain, at least in part, the background responses observed before immunization.

In the present study we showed that bacterial infection of primary B cells and B cell lines with either commensals or enteric pathogens from the *Enterobacteriaceae* family, triggers MAIT cell activation followed by cytokine production. These results support the contention that B cells can function as APC to MAIT cells. This is particularly important because although it has been described that the accumulation of MAIT cells in the gut requires MR1-expressing B cells (8), their role as APC remained unclear. Interestingly, although we found a correlation between expression of MR1 and bacterial antigens on bacteria-infected B cell targets, there were no significant correlations among changes in the expression of MR1 or bacterial expression on bacteria-infected targets and the proportion of MAIT cells producing cytokines. In fact, very low levels of infection observed in targets infected with *E. coli* strain HS induced similar levels of MAIT response as high level-infected targets with *S. typhi*. These results appear to suggest that, like other memory T cells (50), MAIT cells might need low levels of antigenic stimulation to trigger their activation. Alternatively, or concomitantly, we could hypothesize that reaching a specific bacterial antigen load threshold results in B-LCL targets expression of appropriate levels of bacteria-induced B cell antigen(s) sufficient to stimulate MAIT cells. Finally, we cannot discard the possibility that the levels of expression of the key bacterial antigens or MR1 that trigger MAIT activation are not detected by the antibodies used. Taken together, these results suggest that, the quality of MAIT cell responses is more dependent on bacterial load than on MR1 or expression of bacterial antigens on infected target cells. Our observations that a specific bacterial load from different bacterial origins (e.g., *E. coli* strain Nissle or *S. typhi*) was able to generate different levels of infection on the target cells but similar levels of MAIT cells producing cytokines (Figure 5) supports this theory.

Our results using infected targets fixed in 1% paraformaldehyde showed that infected-fixed cells have a reduced ability to trigger MAIT cell activation as compared to live targets. It is remarkable that these results are similar to those reported by Le Bourhis et al. (7) showing that fixed cells resulted in a marked decrease (~70–80% reduction) in MAIT cell activation compared to live cells using different experimental conditions. Le Bourhis et al. (7) reported that MR1 molecules were induced to be overexpressed in fibroblasts and bone marrow-derived dendritic cells prior to bacterial infection as opposed to the natural overexpression of MR1 after infection observed in primary B cells and B-LCL used in our studies, as well as considerably higher MOI’s (up to 1,000:1) as compared with our study (0.3–200). These results support the contention that MR1 in a particular conformation on the cell surface might be needed for optimal MAIT cell activation.

To our knowledge this is the first study uncovering evidence indicating that bacterial load might play an important role in the quality of MAIT cell responses. The specific mechanism(s) by which the bacterial load exert(s) such a strong effect on the quality of the MAIT cell responses remains to be determined. However, it has been demonstrated that BCR-mediated endocytosis allows B cells to concentrate more efficiently smaller than higher amounts of proteins and BCR affinity can also affect the ensuing T cell response qualitatively (28). In fact, Rivera and colleagues have shown that B cells were required for systemic T cell priming when the antigens were present in low amounts than when the antigens were present at higher dose (51). By using transwell experiments in which bacteria-infected B cells and effector cells are separated by a physical barrier, we further confirmed that B cells can function as APC for MAIT cell activation by showing the need of cell-to-cell contact and the inability of soluble antigen alone, in absence of target cells, to stimulate MAIT cells. Previous work has shown that riboflavin metabolites, highly produced during bacteria over growth, might serve as a ligand to MR1 and MAIT cells might use this riboflavin metabolites-MR1 immune complex to detect microbial infection (52). The fact that the results of our transwell filter experiments show that bacteria-infected targets and effector need to be in contact to trigger MAIT cell activation raises the interesting possibility that, as with HLA class I, the loading of MR1 with such metabolites occurs might intracellularly. Further studies using an *E. coli* strain in which enzymes capable of B vitamin metabolism have been genetically inactivated would contribute to answer this important question.

In summary, here we demonstrated that bacterial infection might provide a signal for endogenous MR1 proteins to be expressed on the cell surface. Previous work has suggested this as a possibility but direct evidence had not been provided. Moreover, we confirmed that B cells, both primary and B-LCL, function as APCs for MAIT cells. Finally, we found that MAIT cells have a diminished ability to secrete multiple cytokines than other CD8^+^ T cells. We believe that these novel and unique findings will contribute to a better understanding of the role of MAIT cells on gut immune surveillance.

## Conflict of Interest Statement

The authors declare that the research was conducted in the absence of any commercial or financial relationships that could be construed as a potential conflict of interest.

## Supplementary Material

The Supplementary Material for this article can be found online at http://www.frontiersin.org/Journal/10.3389/fimmu.2013.00511/abstract

Figure S1 **Percentage of MR1-expressing MAIT cells after exposure to uninfected or *E. coli*-infected B-LCLs**. Three different MR1 antibodies were tested: MR1 (clone 26.5) (kindly provided by Dr. Ted H. Hansen), MR1 (goat polyclonal) [Santa Cruz Biotechnology (SC), San Diego, CA, USA], and MR1 (rabbit polyclonal) (GeneTex, Irvine, CA, USA). Data are representative of three experiments.

Figure S2 **Binding of anti-*E. coli* antibodies to the different *E. coli* strains**. *E. coli* strains BL21, Nissle 1917, and HS were directly stained with six serial twofold dilutions (from 3.8 to 120 μg/ml) of anti-*E. coli* antibodies and analyzed by flow cytometry. The results were then fitted in a four parameter logistic curve using Prism software. Shown are median fluorescence intensity (MFI) of *E. coli*-positive B-LCL cells.

Figure S3 **Capture but not only recognition is needed to up-regulate MR1**. B-LCL cells were cultured in medium alone or with lipid A (LPA) (1, 3, and 10 μg/ml) for 2 h, washed, and incubated for an additional 16–18 h in complete media. Cells were then Fc receptor-blocked with human IgG and stained with rabbit anti-MR1 antibodies. After incubation cells were washed and incubated with secondary donkey anti-rabbit-PE antibodies. Cells were then washed, fixed, and analyzed by flow cytometry. **(B)** B-LCL cells untreated or treated with CCD (2.5 μg/ml) were infected with *E. coli* strain BL21 at a multiplicity of infection (MOI) of 1:30 for 2 h and used as positive controls for the LPA experiment **(A)**. **(C)** Uninfected (medium) or *E. coli*-infected B-LCLs (*E. coli*) were stained with normal rabbit serum, followed by secondary donkey anti-rabbit-PE antibodies and used as control (background control). **(D)** *E. coli* strain BL21 stained with rabbit anti-MR1 antibodies was used as an additional control to assess cross-reactivity of the MR1 antibodies with *E. coli*). Numbers correspond to the % positive cells in the denoted quadrants. SSA, side scatter area. Data are representative of one of two replicates with similar results.

Figure S4 ***Listeria* and MAIT cell activation**. Target cells were left uninfected (none) or infected with either *Listeria monocytogenes* (MOI 1:1, 1:10, and 1:50) or *E. coli* strain BL21 (MOI 1:30). THP1, a human monocytic cell line, was used as a target cells. Percentage of MAIT cells producing cytokines (IFN-γ and TNF-α) or degranulating (CD107a/b) was detected after 16–18 h of co-culture with uninfected or infected-B-LCL targets by flow cytometry. Data are representative of two experiments.

Figure S5 **B cells activated MAIT cells in a dose dependent manner**. B-LCL cells were left uninfected (none) or infected with either *Salmonella typhi* (MOI 1:3 and 1:30) or *E. coli* strains HS (MOI 1:30 or 1:100). **(A)** Percentage of B-LCL cells expressing bacterial antigens on their surface were measured by flow cytometry. Targets infected with *E. coli* or *S. typhi* were stained with antibodies to *E. coli* or CSA, respectively. **(B)** Percentage of cytokine-secreting MAIT cells after 16–18 h of co-culture with uninfected or infected-B-LCL targets. Cytokine production by MAIT cells was evaluated by flow cytometry. Data are representative of five experiments.

## References

[1] Salerno-Goncalves R Pasetti MF Sztein MB Characterization of CD8(+) effector T cell responses in volunteers immunized with *Salmonella enterica* serovar Typhi strain Ty21a typhoid vaccine. J Immunol (2002) 169:2196–203 12165550 10.4049/jimmunol.169.4.2196

[2] Salerno-Goncalves R Wyant TL Pasetti MF Fernandez-Vina M Tacket CO Levine MM Concomitant induction of CD4(+) and CD8(+) T cell responses in volunteers immunized with *Salmonella enterica* serovar Typhi strain CVD 908-htrA. J Immunol (2003) 170:2734–41 12594304 10.4049/jimmunol.170.5.2734

[3] Wahid R Salerno-Goncalves R Tacket CO Levine MM Sztein MB Cell-mediated immune responses in humans after immunization with one or two doses of oral live attenuated typhoid vaccine CVD 909. Vaccine (2007) 25:1416–25 10.1016/j.vaccine.2006.10.040 17182155 PMC1840048

[4] Wahid R Salerno-Goncalves R Tacket CO Levine MM Sztein MB Generation of specific effector and memory T cells with gut- and secondary lymphoid tissue- homing potential by oral attenuated CVD 909 typhoid vaccine in humans. Mucosal Immunol (2008) 1:389–98 10.1038/mi.2008.30 19079203 PMC3215293

[5] Salerno-Goncalves R Wahid R Sztein MB Ex vivo kinetics of early and long-term multifunctional human leukocyte antigen E-specific CD8+ cells in volunteers immunized with the Ty21a typhoid vaccine. Clin Vaccine Immunol (2010) 17:1305–14 10.1128/CVI.00234-10 20660136 PMC2944457

[6] Gold MC Cerri S Smyk-Pearson S Cansler ME Vogt TM Delepine J Human mucosal associated invariant T cells detect bacterially infected cells. PLoS Biol (2010) 8:e1000407 10.1371/journal.pbio.1000407 20613858 PMC2893946

[7] Le Bourhis L Martin E Peguillet I Guihot A Froux N Core M Antimicrobial activity of mucosal-associated invariant T cells. Nat Immunol (2010) 11:701–8 10.1038/ni.1890 20581831

[8] Martin E Treiner E Duban L Guerri L Laude H Toly C Stepwise development of MAIT cells in mouse and human. PLoS Biol (2009) 7:e54 10.1371/journal.pbio.1000054 19278296 PMC2653554

[9] Le Bourhis L Guerri L Dusseaux M Martin E Soudais C Lantz O Mucosal-associated invariant T cells: unconventional development and function. Trends Immunol (2011) 32:212–8 10.1016/j.it.2011.02.005 21459674

[10] Daegelen P Studier FW Lenski RE Cure S Kim JF Tracing ancestors and relatives of *Escherichia coli* B, and the derivation of B strains REL606 and BL21(DE3). J Mol Biol (2009) 394:634–43 10.1016/j.jmb.2009.09.022 19765591

[11] Levine MM Bergquist EJ Nalin DR Waterman DH Hornick RB Young CR *Escherichia coli* strains that cause diarrhoea but do not produce heat-labile or heat-stable enterotoxins and are non-invasive. Lancet (1978) 1:1119–22 10.1016/S0140-6736(78)90299-4 77415

[12] Ukena SN Westendorf AM Hansen W Rohde M Geffers R Coldewey S The host response to the probiotic *Escherichia coli* strain Nissle 1917: specific up-regulation of the proinflammatory chemokine MCP-1. BMC Med Genet (2005) 6:43 10.1186/1471-2350-6-43 16351713 PMC1326229

[13] Sheil B Shanahan F O’Mahony L Probiotic effects on inflammatory bowel disease. J Nutr (2007) 137:819S–24S 17311981 10.1093/jn/137.3.819S

[14] Salerno-Goncalves R Fernandez-Vina M Lewinsohn DM Sztein MB Identification of a human HLA-E-restricted CD8+ T cell subset in volunteers immunized with *Salmonella enterica* serovar Typhi strain Ty21a typhoid vaccine. J Immunol (2004) 173:5852–62 15494539 10.4049/jimmunol.173.9.5852

[15] Salerno-Goncalves R Wahid R Sztein MB Immunization of volunteers with *Salmonella enterica* serovar Typhi strain Ty21a elicits the oligoclonal expansion of CD8+ T cells with predominant Vbeta repertoires. Infect Immun (2005) 73:3521–30 10.1128/IAI.73.6.3521-3530.2005 15908381 PMC1111837

[16] Salerno-Goncalves R Sztein MB Priming of *Salmonella enterica* serovar Typhi-specific CD8(+) T cells by suicide dendritic cell cross-presentation in humans. PLoS One (2009) 4:e5879 10.1371/journal.pone.0005879 19517022 PMC2691582

[17] Salerno-Goncalves R Fasano A Sztein MB Engineering of a multicellular organotypic model of the human intestinal mucosa. Gastroenterology (2011) 141:e18–20 10.1053/j.gastro.2011.04.062 21723866 PMC3328095

[18] Lindback T Secic I Rorvik LM A contingency locus in prfA in a *Listeria monocytogenes* subgroup allows reactivation of the PrfA virulence regulator during infection in mice. Appl Environ Microbiol (2011) 77:3478–83 10.1128/AEM.02708-10 21460116 PMC3126465

[19] Nilsson K Klein G Phenotypic and cytogenetic characteristics of human B-lymphoid cell lines and their relevance for the etiology of Burkitt’s lymphoma. Adv Cancer Res (1982) 37:319–80 6305160 10.1016/s0065-230x(08)60886-6

[20] Gordon J Ley SC Melamed MD Aman P Hughes-Jones NC Soluble factor requirements for the autostimulatory growth of B lymphoblasts immortalized by Epstein-Barr virus. J Exp Med (1984) 159:1554–9 10.1084/jem.159.5.1554 6325575 PMC2187288

[21] Tompkins WA Watrach AM Schmale JD Schultz RM Harris JA Cultural and antigenic properties of newly established cell strains derived from adenocarcinomas of the human colon and rectum. J Natl Cancer Inst (1974) 52:1101–10 4826581 10.1093/jnci/52.4.1101

[22] Coren LV Shatzer T Ott DE CD45 immunoaffinity depletion of vesicles from Jurkat T cells demonstrates that exosomes contain CD45: no evidence for a distinct exosome/HIV-1 budding pathway. Retrovirology (2008) 5:64 10.1186/1742-4690-5-64 18631400 PMC2490705

[23] Small PL Isberg RR Falkow S Comparison of the ability of enteroinvasive *Escherichia coli, Salmonella typhimurium, Yersinia pseudotuberculosis*, and *Yersinia enterocolitica* to enter and replicate within HEp-2 cells. Infect Immun (1987) 55:1674–9 3298064 10.1128/iai.55.7.1674-1679.1987 PMC260577

[24] Rubens CE Smith S Hulse M Chi EY Van Belle G Respiratory epithelial cell invasion by group B streptococci. Infect Immun (1992) 60:5157–63 1452349 10.1128/iai.60.12.5157-5163.1992 PMC258292

[25] Altenhoefer A Oswald S Sonnenborn U Enders C Schulze J Hacker J The probiotic *Escherichia coli* strain Nissle 1917 interferes with invasion of human intestinal epithelial cells by different enteroinvasive bacterial pathogens. FEMS Immunol Med Microbiol (2004) 40:223–9 10.1016/S0928-8244(03)00368-7 15039098

[26] Lamoreaux L Roederer M Koup R Intracellular cytokine optimization and standard operating procedure. Nat Protoc (2006) 1:1507–16 10.1038/nprot.2006.268 17406442

[27] Baorto DM Gao Z Malaviya R Dustin ML van der Merwe A Lublin DM Survival of FimH-expressing enterobacteria in macrophages relies on glycolipid traffic. Nature (1997) 389:636–9 10.1038/39376 9335508

[28] Rodriguez-Pinto D B cells as antigen presenting cells. Cell Immunol (2005) 238:67–75 10.1016/j.cellimm.2006.02.005 16574086

[29] Aldemir H Prod’Homme V Dumaurier MJ Retiere C Poupon G Cazareth J Cutting edge: lectin-like transcript 1 is a ligand for the CD161 receptor. J Immunol (2005) 175:7791–5 16339512 10.4049/jimmunol.175.12.7791

[30] Rosen DB Bettadapura J Alsharifi M Mathew PA Warren HS Lanier LL Cutting edge: lectin-like transcript-1 is a ligand for the inhibitory human NKR-P1A receptor. J Immunol (2005) 175:7796–9 16339513 10.4049/jimmunol.175.12.7796

[31] Rosen DB Cao W Avery DT Tangye SG Liu YJ Houchins JP Functional consequences of interactions between human NKR-P1A and its ligand LLT1 expressed on activated dendritic cells and B cells. J Immunol (2008) 180:6508–17 18453569 10.4049/jimmunol.180.10.6508 PMC2577150

[32] Kaper JB Nataro JP Mobley HL Pathogenic *Escherichia coli*. Nat Rev Microbiol (2004) 2:123–40 10.1038/nrmicro818 15040260

[33] Mathias A Longet S Corthesy B Agglutinating secretory IgA preserves intestinal epithelial cell integrity during apical infection by *Shigella flexneri*. Infect Immun (2013) 81:3027–34 10.1128/IAI.00303-13 23753631 PMC3719585

[34] Fenteany G Standaert RF Lane WS Choi S Corey EJ Schreiber SL Inhibition of proteasome activities and subunit-specific amino-terminal threonine modification by lactacystin. Science (1995) 268:726–31 10.1126/science.7732382 7732382

[35] Gottlieb TA Ivanov IE Adesnik M Sabatini DD Actin microfilaments play a critical role in endocytosis at the apical but not the basolateral surface of polarized epithelial cells. J Cell Biol (1993) 120:695–710 10.1083/jcb.120.3.695 8381123 PMC2119548

[36] Perry JW Wobus CE Endocytosis of murine norovirus 1 into murine macrophages is dependent on dynamin II and cholesterol. J Virol (2010) 84:6163–76 10.1128/JVI.00331-10 20375172 PMC2876640

[37] Abos B Gomez Del Moral M Gozalbo-Lopez B Lopez-Relano J Viana V Martinez-Naves E Human MR1 expression on the cell surface is acid sensitive, proteasome independent and increases after culturing at 26 degrees C. Biochem Biophys Res Commun (2011) 411:632–6 10.1016/j.bbrc.2011.07.007 21777569

[38] Shin H Zhang Y Jagannathan M Hasturk H Kantarci A Liu H B cells from periodontal disease patients express surface toll-like receptor 4. J Leukoc Biol (2009) 85:648–55 10.1189/jlb.0708428 19118102 PMC2718806

[39] Ganley-Leal LM Liang Y Jagannathan-Bogdan M Farraye FA Nikolajczyk BS Differential regulation of TLR4 expression in human B cells and monocytes. Mol Immunol (2010) 48:82–8 10.1016/j.molimm.2010.09.008 20956019 PMC2993761

[40] Huang S Gilfillan S Kim S Thompson B Wang X Sant AJ MR1 uses an endocytic pathway to activate mucosal-associated invariant T cells. J Exp Med (2008) 205:1201–11 10.1084/jem.20072579 18443227 PMC2373850

[41] Di Genova G Roddick J McNicholl F Stevenson FK Vaccination of human subjects expands both specific and bystander memory T cells but antibody production remains vaccine specific. Blood (2006) 107:2806–13 10.1182/blood-2005-08-3255 16339400

[42] Nylander S Kalies I Brefeldin A, but not monensin, completely blocks CD69 expression on mouse lymphocytes: efficacy of inhibitors of protein secretion in protocols for intracellular cytokine staining by flow cytometry. J Immunol Methods (1999) 224:69–76 10.1016/S0022-1759(99)00010-1 10357208

[43] Gold MC Lewinsohn DM Co-dependents: MR1-restricted MAIT cells and their antimicrobial function. Nat Rev Microbiol (2013) 11:14–9 10.1038/nrmicro2918 23178389

[44] Chua WJ Hansen TH Bacteria, mucosal-associated invariant T cells and MR1. Immunol Cell Biol (2010) 88:767–9 10.1038/icb.2010.104 20733595

[45] Reijonen H Elliott JF Van Endert P Nepom G Differential presentation of glutamic acid decarboxylase 65 (GAD65) T cell epitopes among HLA-DRB1*0401-positive individuals. J Immunol (1999) 163:1674–81 10415074

[46] Betts MR Brenchley JM Price DA De Rosa SC Douek DC Roederer M Sensitive and viable identification of antigen-specific CD8+ T cells by a flow cytometric assay for degranulation. J Immunol Methods (2003) 281:65–78 10.1016/S0022-1759(03)00265-5 14580882

[47] Walker LJ Kang YH Smith MO Tharmalingham H Ramamurthy N Fleming VM Human MAIT and CD8alphaalpha cells develop from a pool of type-17 precommitted CD8+ T cells. Blood (2012) 119:422–33 10.1182/blood-2011-05-353789 22086415 PMC3257008

[48] Reantragoon R Kjer-Nielsen L Patel O Chen Z Illing PT Bhati M Structural insight into MR1-mediated recognition of the mucosal associated invariant T cell receptor. J Exp Med (2012) 209:761–74 10.1084/jem.20112095 22412157 PMC3328369

[49] McArthur MA Sztein MB Heterogeneity of multifunctional IL-17A producing *S. typhi*-specific CD8+ T cells in volunteers following Ty21a typhoid immunization. PLoS One (2012) 7:e38408 10.1371/journal.pone.0038408 22679502 PMC3367967

[50] Kaech SM Hemby S Kersh E Ahmed R Molecular and functional profiling of memory CD8 T cell differentiation. Cell (2002) 111:837–51 10.1016/S0092-8674(02)01139-X 12526810

[51] Rivera A Chen CC Ron N Dougherty JP Ron Y Role of B cells as antigen-presenting cells in vivo revisited: antigen-specific B cells are essential for T cell expansion in lymph nodes and for systemic T cell responses to low antigen concentrations. Int Immunol (2001) 13:1583–93 10.1093/intimm/13.12.1583 11717199

[52] Kjer-Nielsen L Patel O Corbett AJ Le Nours J Meehan B Liu L MR1 presents microbial vitamin B metabolites to MAIT cells. Nature (2012) 491:717–23 10.1038/nature11605 23051753

