# Supplementary material for: B Cells Modulate Mucosal Associated Invariant T Cell Immune Responses
Source: Front Immunol. 2014 Jan 7;4:511. doi: 10.3389/fimmu.2013.00511 (PMC3882667; doi:10.3389/fimmu.2013.00511)

Supplementary Figure 1

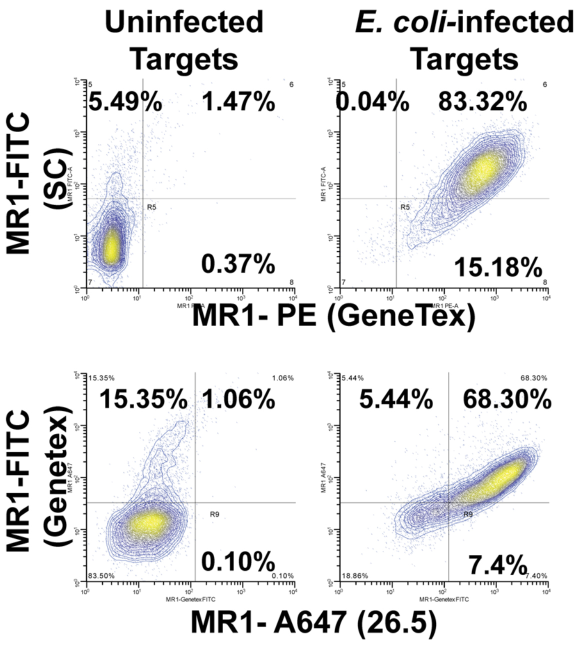

Supplementary Figure 2

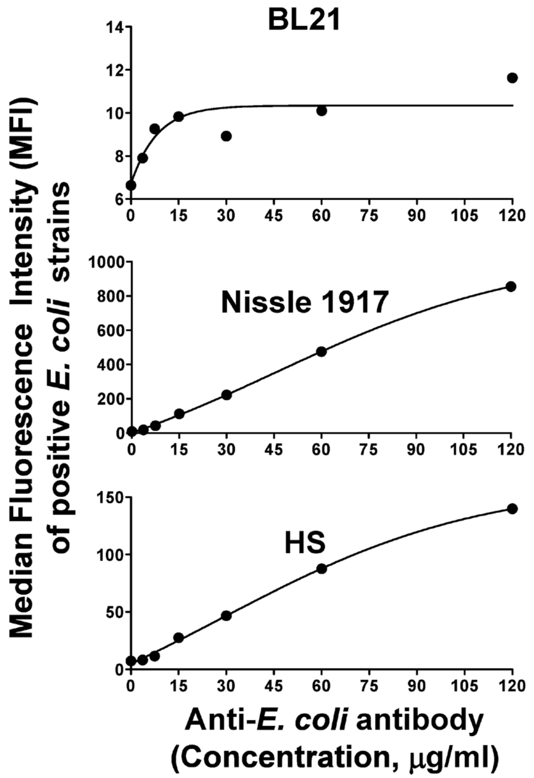

Supplementary Figure 3

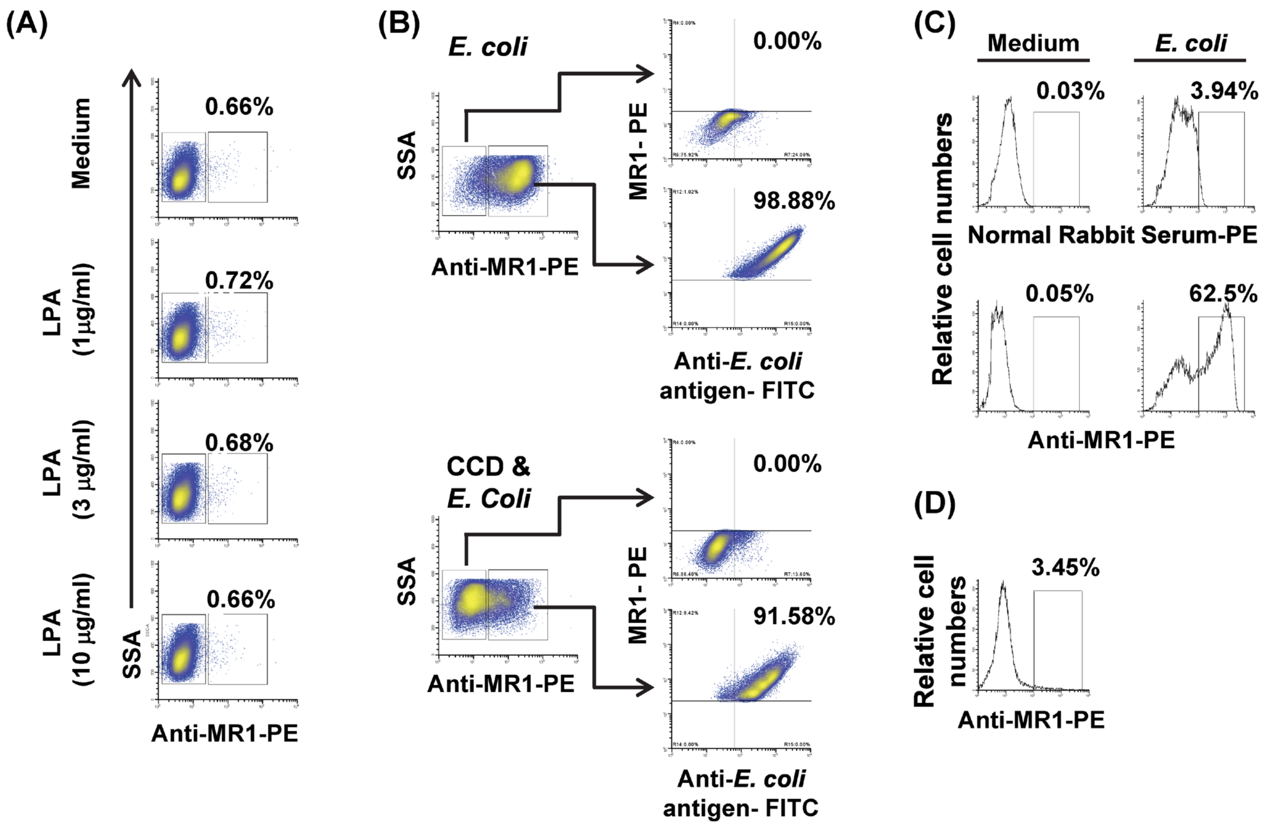

Supplementary Figure 4

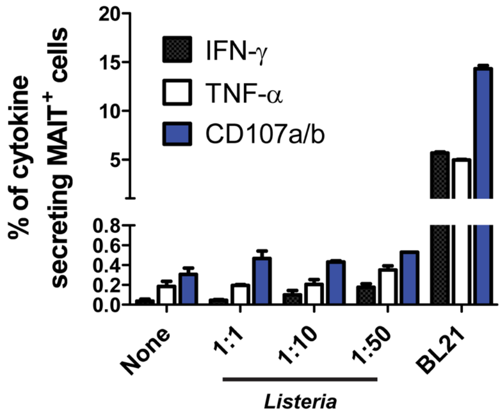

Supplementary Figure 5

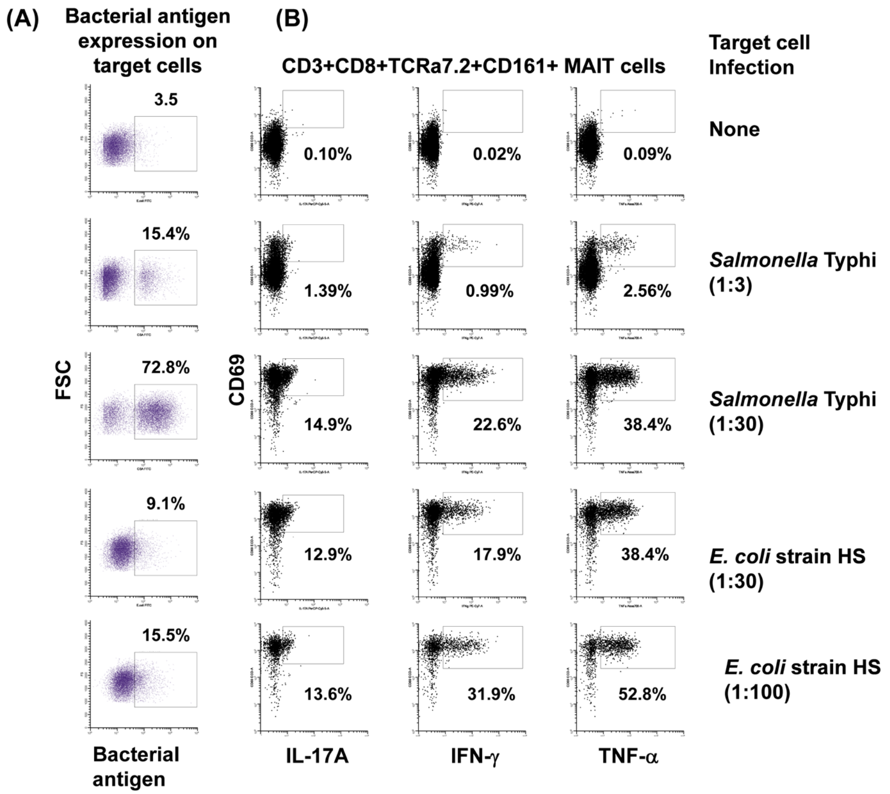

Supplement: Figure S1 — Percentage of MR1-expressing MAIT cells after exposure to uninfected or E. coli-infected B-LCLs. Three different MR1 antibodies were tested: MR1 (clone 26.5) (kindly provided by Dr. Ted H. Hansen), MR1 (goat polyclonal) [Santa Cruz Biotechnology (SC), San Diego, CA, USA], and MR1 (rabbit polyclonal) (GeneTex, Irvine, CA, USA). Data are representative of three experiments. [file 66631_Salerno-Goncalves_Presentation1.PDF]
